# From Polyclonal Sera to Recombinant Antibodies: A Review of Immunological Detection of Gluten in Foodstuff

**DOI:** 10.3390/foods10010066

**Published:** 2020-12-30

**Authors:** Eduardo Garcia-Calvo, Aina García-García, Raquel Madrid, Rosario Martin, Teresa García

**Affiliations:** Departamento de Nutrición y Ciencia de los Alimentos, Facultad de Veterinaria, Universidad Complutense de Madrid, 28040 Madrid, Spain; edugar01@ucm.es (E.G.-C.); ainagarcia@ucm.es (A.G.-G.); raqmad01@ucm.es (R.M.); rmartins@ucm.es (R.M.)

**Keywords:** antibodies, gluten, detection, immunoassays, celiac disease

## Abstract

Gluten is the ethanol-soluble protein fraction of cereal endosperms like wheat, rye, and barley. It is widely used in the food industry because of the physical–chemical properties it gives to dough. Nevertheless, there are some gluten-related diseases that are presenting increasing prevalences, e.g., celiac disease, for which a strict gluten-free diet is the best treatment. Due to this situation, gluten labeling legislation has been developed in several countries around the world. This article reviews the gluten immune detection systems that have been applied to comply with such regulations. These systems have followed the development of antibody biotechnology, which comprise three major methodologies: polyclonal antibodies, monoclonal antibodies (mAbs) derived from hybridoma cells (some examples are 401.21, R5, G12, and α-20 antibodies), and the most recent methodology of recombinant antibodies. Initially, the main objective was the consecution of new high-affinity antibodies, resulting in low detection and quantification limits that are mainly achieved with the R5 mAb (the gold standard for gluten detection). Increasing knowledge about the causes of gluten-related diseases has increased the complexity of research in this field, with current efforts not only focusing on the development of more specific and sensitive systems for gluten but also the detection of protein motifs related to pathogenicity. New tools based on recombinant antibodies will provide adequate safety and traceability methodologies to meet the increasing market demand for gluten-free products.

## 1. Introduction

Gluten is the general term for the ethanol-soluble proteins present in various cereal endosperms, including wheat, rye, barley, spelt, and kamut [1]. The definition by Codex Alimentarius also introduces some physical–chemical concepts: insoluble in water and 0.5 M sodium chloride solution [2]. Currently, this substance is slowly digested and presents a high permanence in the gut. 

In 1924, Osborne introduced a classification method for plant proteins by extraction with different solvents that is still in use. After applying this classification (Table 1), wheat proteins are divided by their solubility behavior into the following fractions: globulins (soluble in a diluted salt solution), albumins (water soluble), gliadins (ethanol soluble), and glutelins (soluble in diluted acetic acid) [3].

Traditionally, gluten proteins have been separated into two fractions that are either soluble or insoluble in alcohol. This division, with some modifications, has remained in use to the present day, with the gluten proteins that are readily soluble in alcohol–water mixtures (e.g., 60–70% ethanol) being called gliadins and those that are insoluble being called glutenins. However, it is now know that the two fractions contain proteins that are structurally related, with the differences in solubility resulting from their presence as monomers that interact by non-covalent forces (gliadins) or as high molecular mass polymers stabilized by interchain disulphide bonds. When present as reduced subunits, the glutenin proteins are also soluble in alcohol–water mixtures and can therefore be defined together with gliadins as prolamins [5]. Glutelins are heterogeneous and can be separated using electrophoresis into over a dozen fractions that as categorized into high molecular weight (HMW) and low molecular weight (LMW) groups [6]. Glutelin subunits have been found to correlate with gluten properties that are related to baking quality [5]. Gliadins are represented as single chain polypeptides, and it is accepted that gliadins are divided, according to their electrophoretic mobility in Polyacrylamide gel Electrophoresis (PAGE) at low pH (lactate-PAGE), into four major groups (α-, β-, γ-, and ω-gliadins, from fastest mobility to slowest) [7]. Gluten proteins contain large repeat domains composed of homologous and repetitive sequences of six-to-eight amino acids rich in proline and glutamine [8]. In addition, when considering the alpha-gliadin structure, their central domain contains the proline- (P) and glutamine-rich (Q) heptapeptide PQPQPFP and pentapeptide PQQPY. This domain contains the most characteristic immunogenic fragment, a 33-mer peptide comprising six overlapping epitopes significant for celiac disease pathogenesis [9], although this peptide is not present in every wheat cultivar [10].

Several fragments of gliadins and glutelins are associated with different types of gluten-related diseases, e.g., α and γ-gliadins in celiac disease [11]; γ-, α/β-, ω5-, and ω1,2-gliadins, as well as HMW and LMW subunits of glutenin, are involved in wheat allergies [12].

## 2. Gluten-Related Diseases

Several diseases related to the exposure to gluten of prone persons that can be classified with etiology into the three main groups of allergy, autoimmunity, and non-celiac gluten sensitivity have been described [13].

Gluten-related allergies, also known as wheat allergies, have a prevalence of 0.1% in the general population [14], and they have developed a well-known two-step pathological mechanism: the sensitization and effector phases [15]. Within this last phase, the onset of the main reactions occurs in minutes to hours after gluten exposure driven by an IgE response. This group includes the following pathologies classified by symptomatology: (a) a respiratory allergy, also known as baker’s asthma, with bronchial symptoms as severe clinical presentation [16]; (b) a food allergy with major digestive presentation [17]; (c) wheat-dependent exercise-induced anaphylaxis (WDEIA), an inflammatory situation triggered by stress [18]; and (d) contact urticaria, with major dermatologic symptoms [14].

The second group of gluten-related diseases is associated with an autoimmune etiology. Celiac disease, with a prevalence of 1% in general population, is the most important [14].

Many molecular mechanisms leading to intestinal damage in celiac disease have been described, although not all have been discovered yet. The ingestion of gluten by sensitized people results in the partial digestion of gliadin (wheat prolamin), which interacts with CXCR3 (chemokine receptor 3) and stimulates the liberation of zonulin [19]. This leads to an increased intestinal permeability, facilitating the translocation of gliadin peptides from lumen to lamina propria. Then, the secretion of innate immunity mediators (Interleukins IL15 and IL8) is triggered, with consequent neutrophil recruitment [20]. The loosen gut barrier facilitates the engagement of toll-like receptor complex 4-M2-CD14 by trypsin and alpha-amylase inhibitors, thus releasing pro-inflammatory cytokines [21]. Following the innate immune-mediated apoptosis of intestinal cells with the subsequent release of intracellular tissue transglutaminase, gliadin peptides are partially deamidated [13]. These deaminated peptides are presented by DQ 2/8 (a class II Major Histocompatibility Complex or HLA cell surface receptor) antigen-presenting cells (APCs) to helper T cells that trigger the maturation and activation of B-cells producing IgM, IgG, and IgA against tissue transglutaminase [22] (for this reason, it is considered an autoimmune disease). Additionally, helper T cells produce pro-inflammatory cytokines like interferon-gamma and tumoral necrosis factor-alpha (TNF-α) [23]. This immune response, together with the function of killer T cells, initiates the enteropathy. Damaged enterocytes express the CD71 transporter to facilitate retrotranscytosis events [24] and further increase intestinal permeability; this spurs a pro-inflammatory and pro-growth environment, resulting in the development of hyperplastic crypts and affecting the absorption of nutrients [13].

In addition to celiac disease, gluten ataxia (a neurological disease [25]) and dermatitis herpetiformis [14] are considered gluten-related autoimmunity diseases. 

Non-celiac gluten sensitivity (NCGS, also denominated non-celiac wheat sensitivity and, sometimes, gluten intolerance) is the third group of gluten-related diseases by etiological classification (non-autoimmune and non-allergic), with a prevalence of up to 7% in the general population [14]. Pathogenic mechanisms are still uncertain, but it seems that innate immunity plays a major role [26]. The signs and symptoms are very similar to other gluten-related diseases, irritable bowel syndrome, and Crohn´s disease. DQ2/8 haplotypes and IgG/IgA anti-gliadin antibodies are present only in 50% of cases. Intestinal damage in this disease is lower than that observed in celiac disease [27].

There has been an intense research into the pharmacological treatment of these diseases, (especially celiac disease) including gluten neutralization agents, disruptors of mucosal transportation or antigen processing enzymes, modifications of the microbiome, immunomodulators, and anti-inflammatory drugs [28]. Notwithstanding, a gluten-free diet is the most recommended and has long been considered the only effective treatment [29]. When gluten consumption is eliminated, the exacerbated immune response is inhibited, leading to the partial (if not complete) healing of the duodenal mucosa along with the resolution of symptoms and signs of malabsorption [30].

## 3. Gluten Content Labeling Legislation in Different Countries

In contrast to other allergens, and following the recommendations included in Codex Standard 118-1979 [2], there is well-developed legislation about gluten presence in food in several countries.

In Europe, the Commission Implementing Regulation (EU) No. 828/2014 of 30 July on the requirements for the provision of information to consumers on the absence or reduced presence of gluten in food [31] rules that “The statement ‘gluten-free’ may only be made where the food as sold to the final consumer contains no more than 20 mg/kg of gluten” and “The statement ‘very low gluten’ may only be made where the food, consisting of or containing one or more ingredients made from wheat, rye, barley, oats, or their crossbred varieties which have been specially processed to reduce the gluten content, contains no more than 100 mg/kg of gluten in the food as sold to the final consumer”.

The U.S. Food and Drug Administration (FDA) has defined the term “gluten-free” for voluntary use in foods that are inherently gluten-free, or when they are not composed of gluten-containing grains—either raw or processed to remove gluten. Any unavoidable presence of gluten in the food must be less than 20 ppm (mg/kg), Food Allergen Labeling and Consumer Protection Act (FALCPA) [32].

Health Canada considers that gluten-free foods are those that contain levels of gluten not exceeding 20 mg/kg as a result of cross-contamination, and they must meet the health and safety intent of B.24.018 (2012). Regarding oats, on 19 May 20, Health Canada registered a marketing authorization 15 that permits the use of gluten-free claims for gluten-free oats [33].

The current legislation in Australia and New Zealand is the strictest. Australia and New Zealand Food Standard Code, standard 1.2.7, states that “for the food to be labeled as gluten-free, the food must not contain: detectable gluten; or oats or their products; or cereals containing gluten that may have been malted, or their products.” For the “not contain detectable gluten” statement, the limit was set at 3 ppm (mg/kg) [34]. 

In Mexico, the executive orders NOM-051-SCFI/SSA1-2010 and NOM-247-SSA1-2008 state that those foods that may produce any kind of allergy and intolerance must be labeled, and those containing grains and derivates must be labeled with “this product contains gluten” statement [35,36]. In Argentina, there is specific legislation for celiac disease (celiac law, passed by the Congress in 2009 (26.588), modified in 2015 (27.196)), claiming this pathology as “disease of national interest,” regulating not only food security issues but also social aspects. A gluten limit of 10 ppm (mg/kg) was set by this law for a product to be labeled as “gluten-free,” including a specific logo. The legislation also applies to medicines [37]. 

In Brazil, Federal Law 8543/1992, mandates that all industrialized foods that contain gluten must carry a warning that they contain gluten. It was updated by Federal Law 10674/2003, determining that all industrialized foods must indicate on their labels and packaging the phrases “Contains Gluten” or “Does Not Contain Gluten.” Brazilian legislation follows the 20 ppm (mg/kg) limit included in Codex Alimentarius [38].

In China, Food Law GB/T23779 issued in 2009 by General Administration of Quality Supervision, Inspection and Quarantine (AQSIQ) “Allergens in prepackaged foods” includes gluten-containing grains and related products amongst the substances that may induce allergic reactions. A 2015 standard specifically applicable to the inspection of gluten allergen ingredients in prepackaged food for export made a clear reference to *Codex* standard STAN 118-1979 in order to verify the compliance of gluten-free claims. This regulation set a maximum limit for a gluten-free claim of 20 mg/kg [39]. However, this regulation does not apply for import or domestic trade.

By Japanese law, the labeling of allergens is designated as mandatory or recommended based on the number of cases of actual illness and the degree of seriousness. To standardize official methods, the Japanese government described the validation protocol criteria in the 2006 official guidelines and stated that any food containing allergen proteins at greater than 10 ppm (mg/kg) must be labeled under the current law [40].

## 4. Methods for Gluten Detection in Food Samples

In recent years, some issues like ongoing legislation, growing general public awareness in food security, and the commercialization of new products focused on a particular population segment (like gluten-free products for celiac patients) has encouraged food industry and research groups to develop more accurate and applicable methods for detecting the traceability of potentially harmful components like gluten.

Nowadays, the gluten detection methods that are widely used in food industry can be classified in two main groups, depending on their target biomolecules: proteins or DNA. 

Methods based on protein detection can be divided into immunological and non-immunological techniques. 

Immunological techniques are based on the high affinity interaction of antibodies and antigens, which has led to the development of different vastly used applications, including:

- ELISA [41]: this is a quick, economic, versatile and robust method. ELISA presents a high sensitivity (in the low ppm range) and optical detection. However, there is a possibility for some false negatives, due to protein denaturalization, and there is some risk of false positives due to cross-reactions with similar but non-target proteins.
- Immunochromatographic assays [42]: these have a visual and simple result interpretation, and they are very simple to use by final operator. Their main weakness is that they cannot quantify.
- Western Blot [43]: this is a highly specific and sensitive method (low ppm), with additional strengths, like confirmatory values (molecular weights) and highly efficient insoluble protein detection. Nevertheless, it is a time-consuming method that must be performed by qualified personnel.

Non-immunological methods are also based on the detection of proteins, but using a mechanism that is completely different to immunological detection. This group encompasses:

- Chromatography methods [44]: these are based on the separation and detection of peptides with a very high sensitivity. The main drawback is that they require complex and expensive instrumentation.
- Mass spectrometry [45]: this is a quick, reproducible, and very precise method of analysis that allows for species detection. However, it is not a quantitative method, and it requires complex and expensive instrumentation.

The other main group of detection methods for gluten is based on the amplification and detection of DNA by the PCR. This technique is highly sensitive (5–50 pg of DNA) and can lead to species identification (useful for cross-contamination studies). Real time PCR methods can be also used for the quantitative detection of gluten when using model mixtures [46,47]. Moreover, PCR methods perform indirect detection (not pathogenic compounds of gluten—rather the DNA that codifies for it) [48].

## 5. Immunological-Based Techniques for Gluten Detection in Food Samples

Immunological techniques are some of the most useful tools for gluten detection. The design of these methods is based in the obtainment of high affinity antibodies or their fragments, guided against noxious parts of gluten. However, though several immunogenic peptides in gluten have been identified, not all have been completely discovered and characterized.

**Figure 1:**
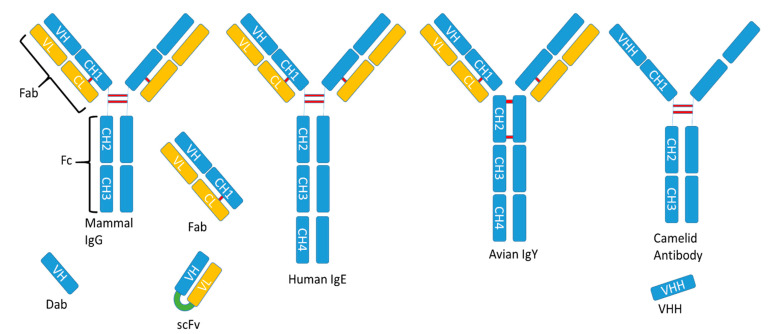
Schematic representation of the structure of antibodies and derived molecules: Ig (Immunoglobulins); Fab (Fragment for antigen binding); Fc (Fragment crystallizable); VH (Variable region of Heavy chain); VL (Variable region of Light chain); CH (Constant regions of Heavy chain); CL (Constant region of Light chain); Dab (single Domain antibody); ScFV (Single chain Fragment Variable); VHH (Variable domain of Heavy chain of Heavy chain antibody).

Antibodies, also called immunoglobulins, are protective proteins produced by the immune system in response to the presence of a foreign substance, called an antigen. Most antibodies used as immune reactants are mammalian IgG. They have a four-chain structure (Figure 1), with two identical heavy chains and two light chains, and they are organized in three functional fractions (two antigen-binding arms, or Fabs, and the fragment crystallizable, or Fc, involved in cell effector systems). The light chain is composed of a constant and a variable region, whilst the heavy chain presents a variable region and a three-domain constant region [49]. Avian antibodies differ from mammals IgG molecules because they have an additional (CH4) domain, like human IgE, while lacking the hinge region that is observed in human IgGs [50]. In addition to these natural structures, many novel antibody fragment molecules have been developed by genetic engineering means, adapting these proteins to different uses. Fabs can be produced alone without attachment to the Fc [51]. Single-chain fragment variable antibodies (ScFvs) are unnatural structures composed of variable regions of light and heavy chains bonded by a flexible linker. Camels are mammals capable to produce a different kind of antibody that lack the light chain (are also known as heavy chain antibodies) and have a heavy chain composed of a variable single domain region (VHH) and two constant domains. Based on this structure, a new type of recombinant fragment consisting only of the VHH domain was developed (Figure 1) [52]. 

Immunoassays have been widely used since mid-20th century. The used antibodies can be classified in two types: polyclonal antibodies, which are obtained by animal immunization, and monoclonal antibodies, which can be obtained by hybridoma-based techniques [53,54] and by a recombinant protein approach.

## 6. Polyclonal Antibodies

**Figure 2:**
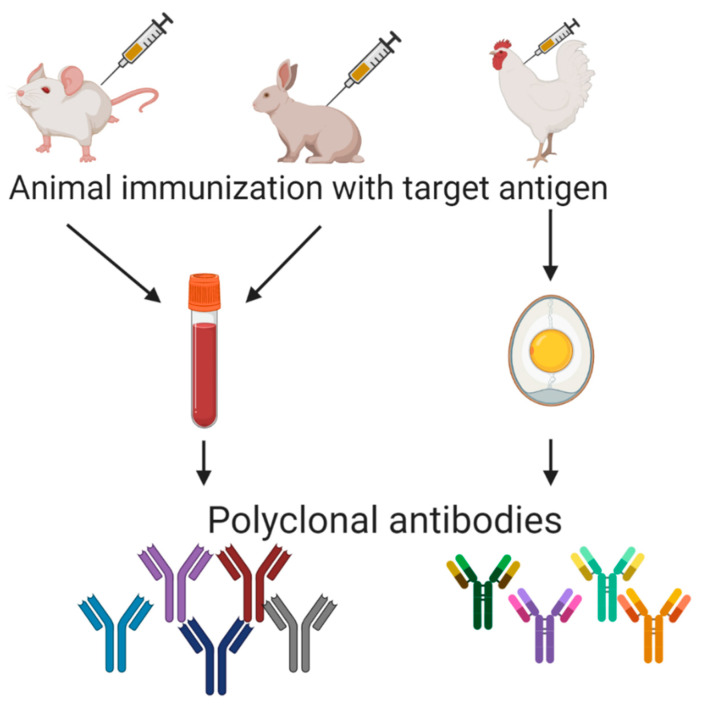
Representation of polyclonal antibodies production.

The first immunoassays were developed thanks to polyclonal antibodies obtained from immunized animals’ sera. The immunization process is well-known (Figure 2), and polyclonal antibodies used to be widely applied. As an example, to obtain polyclonal sera against gluten components in New Zealand white rabbits, emulsified gliadin and glutelin fragments were injected in Freund’s complete adjuvant in the first shot, followed by two further shots of the antigen in Freund´s incomplete adjuvant at two and four weeks later. Good responders (high serum titers) were intradermally given a booster of gliadin, the animals were bled, and antibodies were purified from antiserum by using the ammonium sulfate precipitation method [55]. Though a vast majority of polyclonal antibodies are isolated from mammals (mainly lagomorphs and rodents but also goats and horses), they have also been obtained in chicken embryos (IgY) (Figure 2). These IgYs against gluten have been used not only as reactive for immunoassays but also in prospective therapy for celiac disease intestinal damage [56].

Some examples of commercially available polyclonal antibodies, together with information about the way they have been obtained, can be found in Table 2. 

## 7. Hybridoma Secreted Monoclonal Antibodies

**Figure 3:**
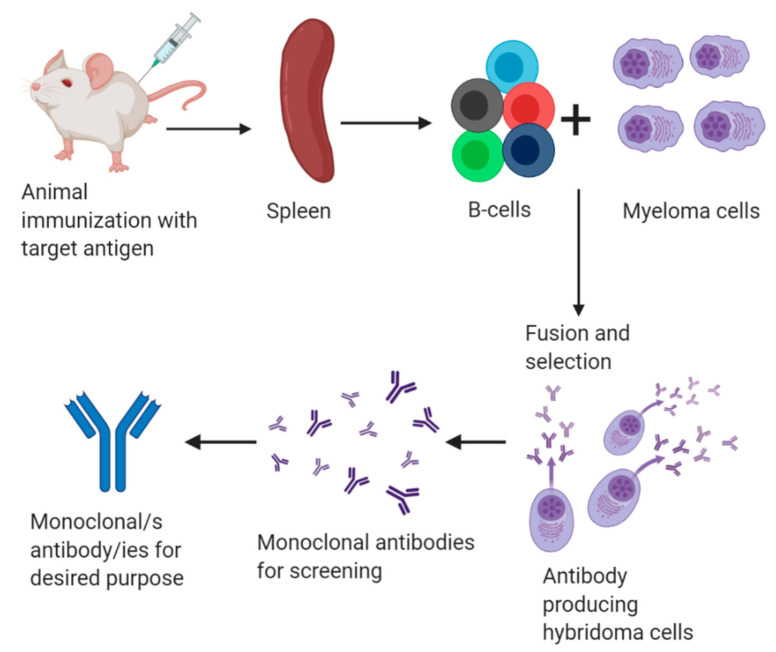
Schematic representation of common monoclonal antibody production.

The research of George Kohler and César Milstein led to the production of monoclonal antibodies secreted by hybridoma cells (Figure 3). This was considered a groundbreaking innovation in many fields like therapeutics and diagnosis [53]. This technology has been also applied to the detection of gluten in foods. The main strength of monoclonal antibodies compared with polyclonal molecules raised in animals is their inter-batch evenness. Additionally, the gold standard method for gluten traceability in food is nowadays based on a monoclonal antibody [33].

One of the earliest developments was proposed by Skerrit and Underwood [57]. A protein fractionation from raw white wheat flour was performed for the immunization of BALB/c mice. Gliadin fractions were prepared by ion-exchange chromatography on sulfo-ethylcellulose to obtain αβ and βγ gliadin pools. Ethanol-precipitated, reduced alkylated glutenin for immunizations was prepared via the extraction of flour with sodium dodecyl sulphate (SDS) and 2-mercapthoethanol. Following the immunization of BALB/c mice with these gliadin and glutelin fractions, spleens were removed and used for fusion with SP 2/0 myeloma cells, and hybridoma cells were prepared and selected using well-established methods [58]. Supernatants from growing cultures were tested for antibodies to either gliadin or glutenins. Positive hybridomas were expanded and re-cloned by limiting dilution. Heavy-chain antibody-isotypes were determined, ultimately obtaining six positive clones from mice immunized with gliadin (five IgG1 isotypes and one IgM), and 11 positive clones from mice immunized with glutelin (nine IgM and two IgG3) [57]. 

A few years later, Skerrit and Hill [59] obtained the monoclonal antibody (IgG1) 401.21, raised against heat-stable ω-gliadins, that recognizes the epitopes PQPQPFPQE and PQQPPFPEE. This monoclonal antibody reacts with ω-gliadins and the corresponding prolamins from rye and barley, as well as with high-molecular-weight glutenin subunits. A sandwich ELISA using the 401.21 antibody was adopted as Official Method 991.19 by the Association of Official Analytical Chemists International (AOACI) [33]. Though this antibody is no longer the basis of the gold-standard, it is currently available in some kits, e.g., the Aller-Tek™ Gluten ELISA assay (ELISA Technologies Inc.) or Veratox© for gliadin (Neogen). 

Sorell et al. [60] developed a sandwich ELISA for gluten analysis in foods using a cocktail of monoclonal antibodies. BALB/c female mice were immunized with wheat, rye, and oat ethanol extracts. Splenocytes of the immunized animals were fused with P3/X63-Ag.653 myeloma cells. Selected hybridomas were grown as ascites in pristane-primed BALB/c mice, and antibodies were purified from ascites by affinity chromatography in a protein A-Sepharose column. Then, seven monoclonal antibodies were characterized (five raised against rye, named R1 to R5, 1 against oats, and one against gliadin, named 13B4). Most of the obtained antibodies displayed a wide cross-reactivity spectrum (R3 showed the highest) with gliadins, hordeins, and secalins. Some of them also cross-reacted with avenins but failed to recognize zeins. Six mAbs were assayed as coating antibodies in the sandwich ELISA using R3 conjugated to Horseradish Peroxidase (HRP) as the labeled antibody. The best results were obtaining using 13B4, which allowed for the selective recognition of gliadins, and R5, which recognized secalins and hordeins. The R5 and 13B4 cocktail, as capture antibodies, and R3-HRP, as detection antibodies, permitted the recognition of gliadins, secalins, and hordeins to the same extent in the range of 3–200 ng/mL, thus improving the results obtained in food samples compared previously available commercial tests and, in many samples, better than mass spectrometry techniques. 

Continuing this work, Valdés et al. [61] developed a novel sandwich ELISA using a single monoclonal antibody (R5) as both the capture (adsorbed) and detection (conjugated to HRP) molecule. A gliadin standard was prepared by ethanol extraction to set up the system, and an aqueous extraction cocktail containing reducing agents was developed for testing samples. The R5 ELISA was able to identify gliadins, hordeins, and secalins with assay sensitivities of 0.78, 0.39, and 0.39 ng/mL, respectively. The detection limit was 1.5 ng gliadins/mL (1.56 ppm gliadins and 3.2 ppm gluten), which was much lower than the existing threshold at that time, with good reproducibility (8.7%) and repeatability (7.7%). These results positioned this system as the best in the field, so it was proposed by the Working Group on Prolamin Analysis and Toxicity (WGPAT) to be included in Codex Alimentarius. 

The sandwich R5 ELISA [61], together with cocktail extraction [62], was validated [63,64] and adopted by the AACCI (American Association of Cereal Chemists International)-approved Method 38-50.01 for gluten detection in corn flour and corn-based products. It was also ratified by the AOACI as Official Method 2012.01 for gluten detection in rice- and corn-based products. This method is also considered by Codex Alimentarius as a type 1 method for the analysis of intact gluten in corn-based matrices. A competitive R5 ELISA was developed for the determination of partially hydrolyzed gluten and accepted as AACCI-approved Method 38-55.01 for gluten detection in fermented cereal-based products [65].

The competitive ELISA method is protected under patent WO2006051145A1, and the extraction cocktail is protected under patent WO2007104825A1. This ELISA and the cocktail extraction method have become the most widely used method for the detection of gluten in food, and they have been commercialized by many companies worldwide. 

Even though the R5-based tests are predominant and official, new methods have been developed in order to fulfill the weaknesses of the established detection methods and growing market demand. 

A new strategy was proposed by Morón et al. [66], because the antibodies that were available in the market recognized peptides of the gluten fraction but were not specifically raised against pathogenic peptides. The identification of the gliadin residues 57–89, which comprise the 33-mer peptide LQLQPFPQPQLPYPQPQLPYPQPQLPYPQPQPF from α-2 gliadin, showed that the highly antigenic gluten epitopes identified to date are in proline-rich regions of gliadin [67]. Due to the low molecular weight of the 33-mer peptide, it required fusion to a carrier protein to enhance the host immune response. Two carrier molecules were used for this purpose: the recombinant heat shock protein *Trypanosoma cruzi* HSP70 and a specific protein fragment derived from *T. cruzi* HSP70, T-HSP70. The hybridomas H-G12 (from B-lymphocytes of mice inoculated with 33-mer-T-HSP70) and H-A1 (from the B-lymphocytes of mice immunized with 33-mer-X2-HSP70) were selected according to the specificity and binding affinity of the antibodies they produced for the 33-mer peptide, as determined by ELISA. Purified monoclonal antibodies were tested against gliadins (commercial sample, reference material, and pepsin digestions) and ethanol-extracted prolamins (from wheat, barley, rye, oat, maize, and rice). Furthermore, a sandwich ELISA was designed using an A1 mAb as the capture antibody and an HRP-conjugated G12 mAb as the detection antibody, with a limit of detection for wheat, barley, and rye of <1 ppm. Moreover, a competitive ELISA based on HRP-conjugated G12 was designed for the detection of the toxic peptide in hydrolyzed food (presenting a limit of detection of <0.5 ppm of gliadin). The recognition sequences were hexameric (QPQLPY) for G12 and heptameric (QLPYPQP) for A1 epitopes. Though the G12 mAb’s affinity for the 33-mer was superior to A1, the A1 mAb presented a higher detection capacity for gluten [68]. 

This work established a valid method concerning accuracy, precision, and reproducibility for the quantification of toxic fractions of gluten that celiac disease patients cannot tolerate. In addition, a slightly greater sensitivity was obtained compared to other commercial antibodies like R5. AgraQuant^®^ (Romerlabs) is a sandwich ELISA method based on the G12 monoclonal antibody approved by the AACCI (Method 38-52.01) [69] and by the AOAC (Official Method of Analysis (OMA) 2014.03) [70] as a certified “method for gluten detection in rice flour and other rice-based products”.

Many studies demonstrated the complexity of celiac disease [67,71,72,73], thus leading to a paradigm shift in the field. The generation of mAbs should not only achieve a very low limit of detection of gluten but also target the determination of disease-inducing peptides. This was the aim of the work by Mitea et al. [74]; they immunized BALB/c mice with synthetic peptides corresponding to known T cell stimulatory epitopes that were coupled to a tetanus toxoid. The target peptides were found in the gliadins glia-α9 (QPFPQPQLPYP), glia-α20 (PFRPQQPYPQP), glia-γ1 (PQQSFPQQQRPFIQPSL), LMW Glt-156 (PPFSQQQQSPFS), and HMW-Glt (PGQGQ(Q/P)GYYPTS(L/Q) QQPQGQQGYYPTSPQQ(P/S)). Because many gluten proteins share a high degree of homology, the authors aimed to prove whether the obtained monoclonal antibodies reacted specifically with only the peptide used for immunization or they detected the other T cell stimulatory sequences. They found that the glia-α20-specific antibody also reacted with the glia-α9 and glia-γ1 peptides. Moreover, the obtained monoclonal antibodies against glia-α9, glia-α20, Glt-156, and HMW-Glt reacted with gluten peptides that are naturally formed during digestion in the gastrointestinal tract, as resulting from the activity of pepsin and trypsin. In addition, all except the LMW-specific antibodies also detected storage proteins in barley and rye, whereas the glia-γ1 specific antibodies also recognized oat proteins. Finally, compared to Ridascreen^®^ Gliadin kit (R5-based), the in-house ELISA for the glia-α9 epitope detected higher gluten concentrations in all analyzed food samples. A competitive ELISA based on the monoclonal antibody anti-glia-α20, called Gluten-Tec^®^, was commercialized by EuroProxima and validated by an interlaboratory study [75], presenting an Limit of Quantification (LOQ) lower than that of the R5 methods (3.6 vs. 5 ppm of gluten, respectively). Monoclonal antibodies were later obtained against the same and different T cell epitopes (glia-γ1, Glt-156, a variant of HMW-gly, and eight peptides). Five antibodies were selected (one anti-α1-gliadin, one anti-γ1-gliadin, two anti-LMW, and one anti-HMW). This method is protected under patent WO2006004394A2.

New challenges appeared after the early 2000s-emergence of severe allergies linked to the ingestion of food products containing even small amounts of hydrolyzed wheat proteins. The main signs and symptoms were found to be WDEIA, anaphylaxis, and urticaria. Sensitization was often related to exposure through cosmetic products. It was described that the triggering ingredient of the mentioned cases of food allergies was the hydrolyzed wheat proteins, where glutamines are converted to glutamic acid by deamidation reactions occurring at high temperature and low pH conditions during industrial processing. Deamination leads to the appearance of novel IgE epitopes, and it was found that prone patients’ sera presented a higher reactivity to deaminated gluten proteins, especially in α, γ, ω_2_, ω_5_, and LMW [76]. 

Regarding this new kind of allergenicity, Tranquet et al. used a new immunodominant neo-epitope (QPEEPFPE derivate of the deamination of QPQQPFPQ) to produce a mAb raised in mice against a peptide that includes the neoepitope [77]. Then, VH and VL were cloned into expression plasmids modified to express IgE-heavy chains to produce a recombinant chimeric IgE [78]. Expression was performed in mammal cells (HEK293). Sera from allergic patients and the recombinant antibody were analyzed by ELISA (with deaminated gluten fractions) and functional basophil assays. It was demonstrated that acid-hydrolyzed wheat proteins that presented higher deamination levels displayed a stronger IgE binding ability and a higher basophil activation capacity. Moreover, the recombinant antibody allowed for basophil degranulation in the presence of deaminated wheat proteins, mimicking patients´ IgE proprieties. This work is an example of another available antibody methodology: the transformation of a “classical” mAb to a recombinant one.

Monoclonal antibodies secreted by hybridoma cells have been proven as a very reliable tool for designing and improving antibody-based gluten detection systems. Some of these developments have been protected by patents, and they are summarized up in Table 3. This research for patent protected monoclonal antibodies and tests for gluten detection was conducted with worldwide.europacenet.com, an international patent database powered by the European Patent Office. Nowadays, most commercially available tests for gluten detection are based on the monoclonal antibodies 401.21, R5, G12, and anti-α20 [33].

## 8. A New Era: Directed Evolution of Recombinant Antibodies

Though the introduction of hybridoma technology led to a great leap forward for the development of antibodies in many fields and forever changed therapeutics and diagnostics, there are still some weaknesses mainly related to the use of animal antibodies for human applications to be solved [79]. In the development of gluten-detection methods, the reliable generation and production of high-affinity antibodies had been achieved thanks to hybridoma technology, and detection limits have been lowered enough to make these antibodies widely used. However, antibodies raised in animals have not presented direct correlation to the human response in many cases (e.g., the humoral response is quite different in a pathological situation, like celiac disease, compared to that produced during animal immunization). Thus, new needs separate from detecting gluten in food samples, like the detection of potentially disease-inductor protein motifs, have appeared. Bio-ethic issues of animal use and sacrifice are also important, and the development of alternatives to the use of animal experimentation is still required [80]. 

In this context, the concept of “directed molecular evolution,” implies making antibodies tailored to, and uniquely suited for, human purposes [81,82]. This concept and its applications rely on the development of genetic engineering and heterologous protein expression. Nevertheless, the molecular evolution of antibodies happens in nature, including human beings, where billions of different antibodies can be produced with three unlinked loci containing the immunoglobulin gene segment [83]. This powerful machine can be replicated and guided to obtain a specific application for gluten detection. The natural machinery of the immune system for making antibodies can be summarized up in five steps: (a) the rearrangement of variable (V) gene segments [84], (b) the surface display of an antibody on a B cell, (c) antigen-driven selection, (d) the secretion of soluble antibody from a plasma cell, and (e) affinity maturation [79]. 

The directed evolution of antibodies needs to overcome two limiting conditions: the generation of enough diversity (referred to as building a library of coding genes for antibody chains), and development of an adequate system for selection and amplification. 

Antibody libraries can be classified into four main groups [85]:

- Immune: constructed based on amplifications of variable (V) genes isolated from immunoglobulin-secreting plasma cells from immunized donors [86].
- Naïve: based on amplifications of V genes isolated from immunoglobulin-secreting plasma cells from non-immunized donors [87].
- Semi-synthetic: derived from unrearranged V genes from pre-B cells (germline cells) or a single antibody framework with genetically randomized complementarity determining regions (CDRs) [88].
- Synthetic: based on a human antibody framework with randomly integrated CDR cassettes [89].

Immune libraries are usually constructed by a two-step cloning (heavy and light chains) assembly PCR method, after mRNA isolation and cDNA preparation from the desired cell type [85]. Naïve, semisynthetic, and synthetic libraries are considered “single-pot” libraries, which means that they can be used for picking an antibody that binds with (almost) every antigen that can be presented [90]. The affinity of antibodies developed from this kind of libraries directly depends on the repertory size RZ (number of different binders within the library). To achieve an affinity in the micromolar (µM) range, an RZ = 10^7^ is required, but an RZ value of 10^10^ is necessary for an affinity of the nanomolar (nM) range. However, immune libraries must be designed and constructed specifically for every single antigen or group of related antigens, and the resulting antibody affinity is driven by their immunogenicity (nanomolar scale is possible with a very immunogenic antigen) [91]. 

The antibody coding genes that conform a library must be cloned in the appropriate vector to develop an adequate system of selection and amplification [85]. Once a library is properly constructed (with enough diversity and cloned in a suitable vector), a process called panning or biopanning (successive rounds of selection and amplification) is performed. For biopanning, it is necessary to express all the antibodies or their fragments included in a library [92]. It has been established that if there are 1–10 binders in 10^7^ clones before selection, there should be 1–10 binders in 10 clones after three-to-five rounds of panning. This process allows for the enrichment of a set of high affinity binders from the library, pairing genotypes (single-strand phagemids that can be “rescued” with a helper phage) with phenotypes (phage-expressing antibodies). 

Peptides, protein domains, and antibody fragments can be displayed for selection and amplification in various ways: phage display [93], in vitro RNA display (divided into ribosome display [94] and cDNA display [95]), and cell surface display [96].

**Figure 4:**
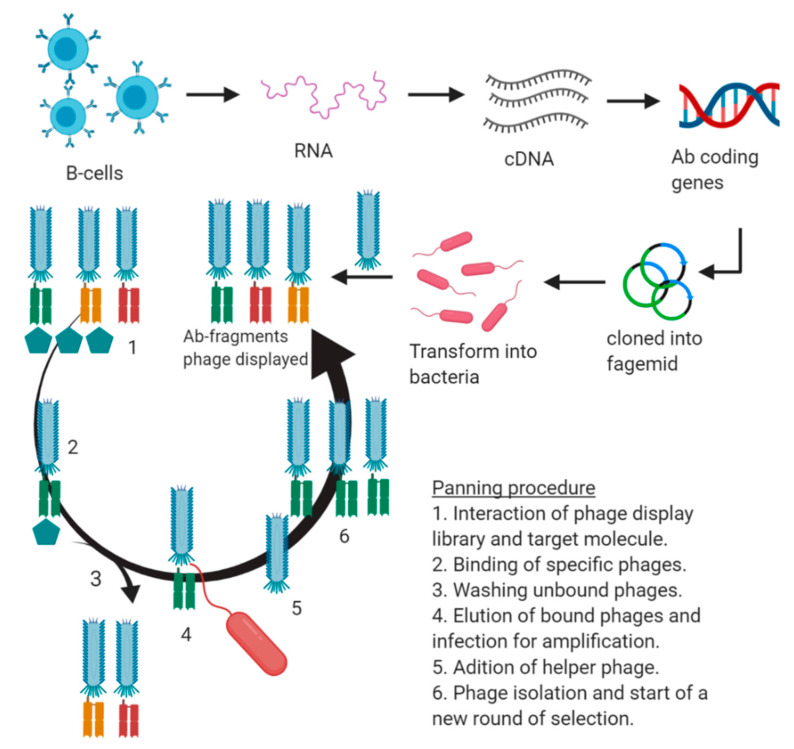
Construction and performance of a directed evolution process mediated of an immune library by phage display.

The phage display of antibody fragments libraries is the most widely used method for the production of recombinant antibodies (Figure 4). The phage display methodology was designed by Smith et al. [97], who introduced gene coding for the bacterial enzyme EcoRI into the gene III of a native bacteriophage and achieved the heterologous expression of EcoRI fused to the phage protein III. Then, a vector called fUSE5 was designed to improve the introduction of exogenous DNA to the filamentous phages and for protein expression [98]. The next step was the development of the affinity selection procedure: (a) the immobilization of a selector, (b) the addition of input virions (with the displayed peptides that bind the selector), (c) the washing away unbound virions, (d) the release of bound virions, and (e) the amplification of the released virions by infecting a proper bacteria [93]. 

The first application of phage display was the epitope mapping of a given antibody [93,99]. There have been huge developments of this system with different applications, although the methodological basis has not significantly changed since Smith and collaborators achieved the proof of concept [93]. 

New vectors have been developed with some common features: a double replication origin (f1 or similar for protein phage fusion and an *Escherichia coli* ori), a selection marker (usually an antibiotic resistance gene), and binder-coding genes (usually peptides, protein domains, or antibody fragments) fused to a phage protein (usually capsid proteins) [100]. 

Vectors can be classified to several types according to their design. If the binder is fused to phage gene III, it will be expressed fused to protein III (up to five copies), constituting a type 3 phagemid. However, if it is fused to gene VIII, it will be expressed fused to protein VIII (up to 2400 copies), constituting a type 8 phagemid. Types 33 and 88 follow the same concept, but they have two copies of either gene III or VIII. Types 33 and 88 are also phagemid-based, but the virus particles are only formed in cells carrying the phagemid genome when they are infected with the helper phage virus [101]. Though pIII and pVIII-based vectors are the most frequently used, there have been some attemps to clone binders in the VII and IX genes, which are minor capsid proteins [102]. 

**Figure 5:**
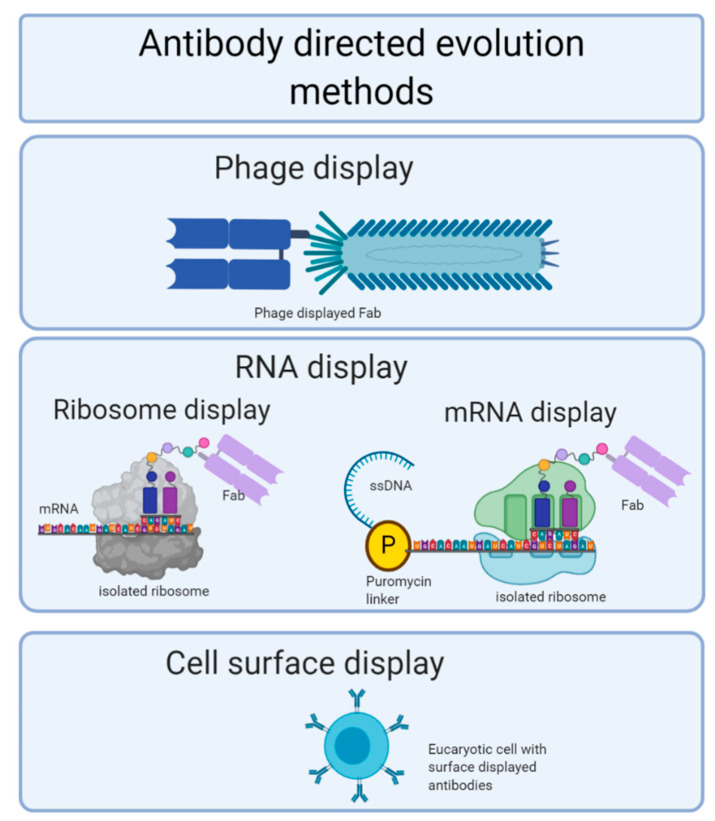
Schematic representation of antibody-directed evolution methods.

Due to the variability of culturing cells, new cell-free methods have been developed with two main variants: ribosome and mRNA (or cDNA) displays (Figure 5). The ribosome display is performed as follows: a DNA encoding library is in vitro transcripted to a single strand mRNA lacking stop codon, then it is in vitro translated to obtain a native protein attached to a ribosome, and affinity selection is performed. Then, mRNA is released from the ribosomes and reverse transcripted to single strand DNA that can be replicated and/or mutated, thus becoming the base of another round of selection [103]. mRNA display improves the coupling between genotypes and phenotypes. The process starts with the transcription of a DNA library; then, mRNA is ligated to a DNA linker connected to puromycin. The in vitro translation of this complex allows for a peptidyl transferase reaction that results in a covalently linked mRNA–protein complex that has the puromycin-linker-mRNA. Then, the mRNA is retrotranscribed, and affinity selection is performed. Finally, high-affinity complexes are eluted, releasing only DNA strands by RNA hydrolysis. This DNA can be mutated and is the starting material for the subsequent round [94]. A cell-surface display (Figure 5) allows peptides and proteins to be displayed on the surface of cells by fusing them with anchoring motifs [96] instead of using bacteriophages. Protein expression improves by using more complex organisms like Gram-negative bacteria [104], Gram-positive bacteria [105], yeasts [106], and mammalian cells [107].

Directed-evolution methods have become groundbreaking technologies for antibody development at a similar level to hybridoma-derived monoclonal antibodies. In the field of gluten-related diseases, they have been applied with two main objectives. Most of the published works have been focused on using a phage display as a tool for celiac disease molecular characterization. The other significant approach is to apply recombinant antibodies to develop gluten-detection systems, because hybridoma antibodies were previously used. In the context of the molecular characterization of celiac disease, phage display technology has mainly been used to develop high-affinity antibodies against human tissue transglutaminase, an autoantigen with a major role in celiac disease [108].

An early strategy by Sblattero et al. [109] was to study the immune response against tissue transglutaminase by building an immune library from the peripheral blood lymphocytes (PBLs) of a patient with celiac disease and performing a selection against four related antigens (α-gliadin, β-lactoglobulin, human tissue transglutaminase, and guinea pig transglutaminase). Some polyreactive and monoreactive antibodies were obtained against the first two antigens but not to transglutaminase. VH4 was the main family of the anti-α-gliadin selected antibodies. Following this approach, Mazari et al. [110] produced and analyzed six immune ScFv libraries from peripheral and intestinal lymphocytes (IBLs) collected from three celiac patients, and they concluded that intestine-derived antibodies from all selected patients recognized human transglutaminase (with a bias toward the use of the VH5 family), whereas peripheral blood-derived antibodies recognized α-gliadin. Following this research, Sblattero et al. [111] built up an ScFv library based on the amplification of the two VH5 family genes from intestinal lymphocytes, resulting in a rapid characterization of the anti-transglutaminase response that could be applied in asymptomatic patients whose serum antibodies may be undetectable.

Phage display has been revealed as a very reliable method for celiac immunity characterization. Not et al. [112] generated an immune library by amplifying the VH5-51 gene from bowel biopsy specimens of 22 relatives of celiac patients and analyzing its interaction with human transglutaminase. They found that genetically predisposed individuals to celiac disease produce VH5 anti-transglutaminase intestinal antibodies (anti-TG2) in the absence of serum anti-TG2 antibodies. 

Another use of phage display libraries for the characterization of celiac disease was the work of Hoydal et al. [113], who investigated the antigen presentation process during mucosal immune response. They applied a large naïve human ScFv library for the isolation of specific binders against the complex HLA-DQ2.5:DQ2.5-gliadin-α1a. Then, the obtained antibodies were applied to cells from intestinal biopsies from patients with celiac disease, which allowed for the identification of plasma cells as the most abundant gluten peptide-MHC-expressing cells in inflamed intestinal tissues from celiac patients. 

Rhyner et al. [114] reported the construction of three unique isotype ScFv libraries (IgA, IgG, and IgM) from a celiac patient and a healthy control individual, and they demonstrated that all libraries from the celiac patient, but none from the control donor, were selectively enriched in gliadin-binding phage clones after four rounds of biopanning. This method not only resulted in a suitable approach to obtain high-affinity antibodies against gliadin but also allowed for the isotype-specific characterization of the immune responses occurring in a pathological condition.

Phage display technology can be also used as a scanning method to study biological interactions when conducted with peptides libraries. An example of this concept in celiac disease was developed by Chen et al. [115]; a random peptide phage display library was enriched in gliadin-binding peptide-phages by several panning cycles against immobilized gliadin proteins. Several peptide-expressing phage clones were able to inhibit the interaction between gliadin and anti-gliadin antibodies. Moreover, the 12-mer peptides encoded by selected clones were synthetized in vitro and analyzed in competition experiments that revealed the binding of different peptides to different sites of the gliadin. The authors suggested the potential use of these peptides to detoxify gluten.

All mentioned works (summarized up in Table 4) are examples of the potential of phage display as a tool for celiac disease molecular characterization. Increased understanding of celiac disease has led to better diagnosis and prevention measures, like those derived from the discovery of the major role of tissue transglutaminase and toxic components of gluten. 

Though most of the published phage-display applications related to gluten have been focused on studying celiac disease antibody response, there is a growing interest in the development of recombinant antibodies for the detection of gluten in foodstuffs as a final application (Table 5). 

Doña et al. [116] developed a system for gluten detection in food samples based on VHH antibodies. A VHH phage display library was constructed from PBLs isolated from gliadin-immunized llamas, and the selection of gliadin-binding VHH was performed under denaturing conditions. The selected VHH allowed for the development of a capture ELISA system (using gliadin-binding VHH as capture and anti-gliadin mouse-derived monoclonal antibody for detection) able to detect gliadin in samples that tested negative with other ELISA kits. However, the method was only applicable to wheat gluten detection, as the selected VHH did not react to barley or rye prolamins. 

García-García et al. [117] designed and developed a phage-ELISA method for gluten analysis in foodstuffs based on single-domain antibody (or dAb) fragments using a semi-synthetic library developed by Christ et al. [119]. The library was enriched in high-affinity dAbs by successive rounds of selection against the consensus peptide CPFPQQQPFPQQPFPQQQPFQQQPFQQPFQQQPQQQP [120] that includes epitopes that can be found repeatedly in gluten proteins. A phage ELISA method was used to screen 50 commercially available food products, with a limit of detection of 20 ppm of gluten. The method was able to ascertain compliance with the labelling of gluten-free products. Moreover, this work completed an animal-free antibody developing process applicable to the detection of gluten in foods.

Phage display is not the only directed evolution technique that has been applied in gluten research. Jayathilake et al. [118] demonstrated the application of a cell-free display method in this field. A VHH cDNA library was developed from alpaca lymphocytes to pair genotypes (cDNA) and phenotypes (VHH) thanks to a puromycin linker. Several rounds of selection were performed against immobilized gliadin to obtain three high-affinity binders that were used as the basis of a novel gluten detection technique called cDNA display mediated immuno-PCR (cD-IPCR) [121]. The cD-IPCR method was able to detect very low gliadin concentrations (0.001–10 µg/mL) in food. This method can be improved with the objective of obtaining effective binders for toxic and complex proteins, which is difficult to achieve using conventional methods. 

VHH-, dAb- and cD-IPCR-based methodologies have become pioneers for a new generation of gluten traceability systems. 

## 9. Final Remarks

Nowadays, the detection and traceability of allergens and related substances (like gluten) has become one of the hottest topics in the food and drink industry due to the following issues: (a) consumer demand for accessible, clear, and accurate label information; (b) growing health and nutrition social concerns; (c) an increasing prevalence of food-related diseases (as a result of better diagnosis and knowledge on their pathogenesis); and (d) the consumption of new foods and ingredients derived from a competitive and globalized market. 

This situation is a continuous challenge for food science that has demanded the design and development of new gluten traceability systems that can work with changing and stricter regulated limits of detection and quantification. New developments will be focused not only on lowering gluten detection limits but also on identifying those components that are able to trigger gluten-related diseases. 

Improving the immunodetection of gluten in foods depends on the obtention of better antibodies. Chronologically, the strategies used for developing these antibodies can be summarized as follows: (1) polyclonal antibodies raised by animal immunization comprised the first method that is still currently in use, with two main variants: mammal-derived IgG and chicken-derived IgY; (2) monoclonal antibodies like 401.21 and R5 were produced from hybridomas raised against different cereal protein extracts; (3) monoclonal antibodies like G12, produced from hybridomas, were raised against recombinant proteins that were implied in celiac pathogenesis; and (4) recombinant antibodies were obtained by directed molecular evolution with phage or cDNA display technology. Each of the technologies outlined in this review (polyclonal, monoclonal, and recombinant antibodies) has advantages and disadvantages (Table 6). The selection of the appropriate methodology will depend on the intended use and resources available. 

Future perspectives for this field include the discovery of new antibodies, with an increasing affinity for disease triggering gluten peptides. For such a purpose, it is necessary to continue to unveil the molecular mechanisms of gluten-related pathologies, like celiac disease (the most widely studied) and others, particularly non-celiac gluten disease, that present the highest prevalence.

An integrative strategy is currently being applied. Building antibody libraries from gluten-sensitive patients should allow for the study of immune response against gluten and the production of recombinant antibodies with the same “behavior” as those found in patients, thus making it possible to detect those protein motifs that are dangerous for prone people in foods.

Additionally, there has been intense research into new devices that unify gluten extraction and antibody-mediated detection, as well as the development of easier and faster analysis methodologies that can move from laboratory applications to consumer-friendly devices to be used in homes or restaurants.

Preventing gluten-derived food risks requires of a multi-disciplinary approach that implies basic and clinical research, biotechnological and engineering developments, and a farm-to-table philosophy; these goals will only be reachable with the help from all value-chain members, from raw material producers to final consumers. 

## Figures and Tables

**Table 1:** Protein fractions from cereal grains [4].

Osborne Fraction		Wheat	Rye	Oats	Barley	Corn
Globulin		Edestin				
Albumin		Leucosin				
Gluten	Prolamin	Gliadin	Secalin	Avenin	Hordein	Zein
	Glutelin	Glutenin	Secalinin	Aveninin	Hordeinin	Zeinin

**Table 2:** Some examples of polyclonal antibodies against gluten or its fragments.

Antibody	Company	Host	Isotype	Raised against
PAB29118	Abnova	Chicken	IgY	Wheat flour protein extract
MBS617177	MyBioSource	Rabbit	IgG	Wheat gluten
MBS838918	MyBioSource	Rabbit	IgG	Wheat gliadin
MBS625849	MyBioSource	Chicken	IgY	Wheat gluten
LS-C66756	LifeSpanBiosciences	Rabbit	IgG	Wheat gluten
LS-C129350	LifeSpanBiosciences	Chicken	IgY	Wheat gluten
LS-C750830	LifeSpanBiosciences	Chicken	IgY	Wheat gluten
G8138-01	USBiological	Rabbit	IgG	Wheat gluten
G8138-02	USBiological	Chicken	IgY	Wheat flour protein extract
AS09 571	Agrisera	Chicken	IgY	Wheat flour protein extract
PA5-97536	Invitrogen	Rabbit	IgG	Wheat gliadin native protein
G9144	Sigma-Aldrich (Merck)	Rabbit	IgG	Native and heat-treated wheat gliadin

**Table 3:** Several examples of registered patents claiming monoclonal antibodies and antibody-based methods related to gluten detection.

Patent No.	Summarized Patents	Applicant
WO2006004394A2	A method for the screening of basic ingredients, semi-manufactured ingredients, and food products that are intended to be used in a gluten-free diet, based on antibodies raised against T cell stimulatory peptides.	Academisch Ziekenhuis Leiden (The Netherlands)
WO2006051145A1	Competitive ELISA for the detection of gluten hydrolysate based on the R5 monoclonal antibody.	Consejo Superior de Investigaciones Científicas (Spain)
WO2007104825A1	Method for extracting gluten from processed (by heat) and unprocessed foods based on the use of ionic and non-ionic detergents as prior step for ELISA tests.	Consejo Superior de Investigaciones Científicas (Spain)
WO2014132204A1	Monoclonal antibody that is capable of bonding to deamidated gluten proteins (related with celiac disease pathogenesis) and has no cross-reaction with non-deamidated gluten proteins.	Institut national de la recherche agronomique (France)
WO2015164615A1	Isolated monoclonal antibodies and fragments that bind to 11 peptides that can be found in gluten proteins.	University of Chicago (USA) and University of Oslo (Norway)
WO2018071718A1	Antibodies, fragments, or polypeptides in the detection of gliadin: heavy chain and light chain variable sequences, and associated sequences of complementarity-determining regions (CDRs).	Nima Labs Inc. (USA)
WO2019154559A1	Immunoassay methods for the quantification of the total gluten content of grains in food samples.	R-Biopharm AG. (Germany)
ES2392412A1	Solutions for the extraction and solubilization of gluten, composed of arginine and ethanol.	Biomedal S.L (Spain)
GB2207921A	Hybridoma cell line ATCC HB9798 that produces monoclonal antibodies directed against omega gliadin protein of wheat and related proteins in rye and barley.	Commonwealth Scientific and Industrial Research Organization (United Kingdom)
CN101698832A	Anti-gliadin monoclonal antibody and the hybridoma cell line obtained from it.	Quingdao Biomade Technology Company Ltd. (China)
CN107860918A	Colloidal gold immunochromatography test strip for the gluten allergen in food and the preparation method of colloidal gold immunochromatography test strip.	Biofront Technology Company Ltd. (China)

**Table 4:** Several examples of phage display technology applied to celiac disease research.

Type of Library	Antibody Format	Isotype VH Family Gene/s	Selection Driven by	References
Immune (peripheral blood lymphocytes (PBLs) from celiac patients)	Single-chain fragment variable antibody (ScFv)	IgG VH4	α-gliadin human transglutaminase Other antigens	[109]
Immune (PBLs and intestinal lymphocytes (IBLs) from celiac patients)	ScFv	VH5	Human transglutaminase α-gliadin	[110]
Immune (IBLs from celiac patients)	ScFv	VH5	Human transglutaminase	[111]
Immune (IBLs from celiac patient relatives)	ScFv	VH51-1	Human transglutaminase	[112]
Naïve	ScFv	NA	Gliadin HLA-presenting peptides	[113]
Immune	ScFv	IgA, IgG, and IgM	Gliadin	[114]
Random peptides	Random peptides	NA	Gliadin HLA-presenting cells	[115]

**Table 5:** Several examples of directed evolution methods for the obtention of antibodies for detection of gluten in foods.

Technology Used	Type of Library	Antibody Format	Selection Driven by	Gluten Detection Method	References
Phage display	Immune	VHH	Gliadin	Capture ELISA	[116]
Phage display	Naïve	single-domain antibody (dAb)	Consensus gluten peptide	Phage ELISA	[117]
cDNA display	Naïve	VHH	Gliadin	cDNA display mediated immuno-PCR (cD-IPCR)	[118]

**Table 6:** Comparison of technologies available for production of antibodies for gluten detection in foods.

Technology	Market Available Test for Gluten Detection Based on These Technologies	Validated Tests for Gluten Detection Based on These Antibodies	Clinical Applications Developments with These Antibodies	Inter-Batch Evenness	Need Animal Experimentation for Its Development	Technical Readiness
Polyclonal antibodies	Yes	Yes	Yes	Variable	Yes	Mature
Monoclonal antibodies	Yes	Yes	Yes	Yes	Yes	Mature
Recombinant antibodies	Not yet	Not yet	Yes	Yes	Not all	Recent
